# Case Report: Parental Loss and Childhood Grief During COVID-19 Pandemic

**DOI:** 10.3389/fpsyt.2021.626940

**Published:** 2021-02-11

**Authors:** Susana Santos, Teresa Sá, Inês Aguiar, Inês Cardoso, Zulmira Correia, Teresa Correia

**Affiliations:** Department of Child and Adolescent Mental Health, Centro Materno-Infantil Do Norte, Centro Hospitalar e Universitário Do Porto, Porto, Portugal

**Keywords:** child and adolescence psychiatry, parental death, COVID19, bereavment, childhood grief

## Abstract

The coronavirus disease 2019 (COVID-19) pandemic is an unprecedent public health crisis, transforming many aspects of our daily life. Protection measures, such as social distancing, nationwide lockdowns, and restrictions on hospital visits and funerals have a serious impact on how people mourn their loved ones. The grieving process during childhood and adolescence evolves along the developmental stages and is a dynamic, non-linear process that needs time. Parental death increases the risk for psychopathology in the short and long term. We present a case of an 11-year-old girl referred to child psychiatry-liaison service by her neurologist due to peer relationship problems and sadness. Fifteen days before her first psychiatric consultation, her father suffered a myocardial infarction complicated with hypoxic ischemic encephalopathy, and he was hospitalized in the intensive care unit. Positive coping mechanisms and adaptive emotional expression strategies were explored during her consultations. Her father died 2 weeks after emergency state and nationwide lockdown was declared in Portugal, during the first COVID-19 outbreak. The family did not have the opportunity for a proper farewell, the funeral obeyed strict rules, and the patient and her family were at home, due to social distancing and school closure policies. Consultations were maintained by telephone calls and, less frequently, by face-to-face appointments. Adaptive and helpful strategies to grieve were shared with the patient and her mother. Intervention with the mother alone was also helpful. Death circumstances related to COVID-19, confinement policies, and social–economical stressors can intensify the grief experience, increasing the risk for complicated grief. Although psychiatric teleconsultation is essential during COVID-19 pandemic, it poses various limitations. Non-verbal communication clues may not be totally apprehended; it may represent a problem in the therapeutic relationship, and access to technology can be difficult for psychiatric patients and clinicians. COVID-19 pandemic policies should include mental health protection measures, which should facilitate adjusted grief responses for those who lose a loved one during this pandemic.

## Introduction

The coronavirus disease 2019 (COVID-19) pandemic represents a challenge at multiple levels. It is an unprecedented public health crisis with undeniable social, economic, and emotional impact.

The fear and uncertainty along with dramatic changes in our daily lives imposed by physical and social isolation, home schooling, and telework strain families' lives and may have a negative impact on children and adolescents' mental health (1). In a cross-sectional study among 8,079 Chinese adolescents conducted by Zhou et al. during the COVID-19 outbreak, the prevalence of depressive and anxiety symptoms was 43.7 and 37.4%, respectively. More than 30% of the adolescents reported combined symptomatology. Being female, in senior high school, and having low levels of awareness of COVID-19 were related to higher prevalence of depressive or anxiety symptoms (2). Duan et al. applied online questionnaires to 359 Chinese school-age children and 3,254 adolescents during COVID-19 outbreak and reported high anxiety levels in 23.9% of children and in 29.27% of adolescents. Prevalence of clinical depressive symptoms was 22.28% (3). The pandemic situation can also worsen previous psychiatric disorders, as Nissen reported regarding a sample of children and adolescents diagnosed with obsessive–compulsive disorder (4).

Fear of death may be exacerbated in this period. How children and adolescents understand death and mourn a loved one depends not only on their development stage but also on religion and social aspects, cognitive ability, and prior life events.

Infants and toddlers react to separation from an attachment figure with distress. Due to their sensitivity to routine changes, routines should be maintained as much as possible. Preschool children do not understand death as irreversible, and their characteristic magical and egocentric thinking may lead them to believe they somehow cause the death with their actions, words, or thoughts, inducing guilt and regret. As they do not understand death as irreversible and non-functional, they may believe that the deceased will return or the deceased did not cease to sleep, eat, and feel. They often cope through play, which can be misinterpreted by adults. As children enter school age, they progressively develop logical, concrete operational thinking. They can understand death's irreversibility and may ask specific and scientific questions about death and its circumstances. Their thinking is less egocentric than younger children. Therefore, besides being concerned about their own needs, they also worry about others' well-being. As adolescents develop existential and abstract thought, they can understand death as irreversible, universal, and non-functional, so their mourning process resembles that of the adults. Structural developmental tasks of adolescence (identity consolidation, separation from family, and identification with peers) can be at risk when a family member dies, as they may feel different from their peers. Depending on their copying style, they may be at risk of developing internalized or externalized perturbed responses to death (5, 6).

All children and adolescents go through a mourning process when someone important dies, and it includes a wide range of emotions, cognitions, physical symptoms, and behavioral changes (6, 7). *On death and dying*, Kubler-Ross characterized grief as a five-sequential-stage process: denial, anger, bargaining, depression, and acceptance (8). In the last decades, other models have been presented, understanding grief as a dynamic process that implies proactivity in adaptation to the loss. According to Worden, grief process develops through four tasks: accepting the reality of the loss, processing the pain of grief, adjusting to a world without the deceased, and finding a way to remember the deceased through life (6). As one of the most personal experience someone lives, grieving takes its own time in a fluid, non-linear process, where tasks are revisited and reexperienced through life. Persistence of grief response and failure to adapt to the loss, with intrusive thoughts, avoidance behaviors, and loss of interest in daily activities are signs of a maladaptive grief response. Complicated grief was previously described in children and adolescents and is associated with increased risk for depression and functional impairment (7, 9). The 11th edition of the International Classification of Diseases introduced the diagnosis of Prolonged Grief Disorder when it persists for an abnormally long period of time following the loss (more than 6 months) (10). The 5th edition of the Diagnostic and Statistical Manual (DSM) of Mental Disorders considers a specific set of bereavement-related symptoms (Persistent Complex Bereavement Disorder) under the diagnosis of Other Specified Trauma and Stressor-Related Disorder. This diagnosis, which can only be made 12 months (6 months in children) after the loss, is nowadays included in Section III (Proposed disorders for future study) of DSM (11). Despite its specificity to detect prolonged grief reactions in children, these criteria are not sensitive enough in children, as pointed out in a study conducted by Melhem et al. (12). The addition of a new diagnosis, “Prolonged Grief Disorder,” to the Depressive Disorders Chapter in Section II of DSM was recently proposed, including symptoms of identity disruption, marked sense of disbelief about the death, avoidance of reminders of the loss, difficulty moving on with life, intense emotional pain or emotional numbness, and intense loneliness (13).

It is of consideration that the internal resources someone has and develops to integrate the loss and reconstruct life are as important as the time grief may take. As Worden says, “healing comes from what the grieving person does with the time” (6). In children and adolescents, those resources are inherently changing along with developmental stages. Therefore, they may revisit the grief as they grow, integrating it differently and giving it new meanings, in a developmentally appropriate way (14).

## Clinical Report

We present a case of an 11-year-old girl, referred to the child psychiatry-liaison service by her neurologist due to peer relationship problems and sadness. The patient had no prior personal or familiar psychiatric history. She was diagnosed with centrotemporal spikes epilepsy 2 years before and was prescribed with prednisolone, valproate, and clobazam. Her last intelligence assessment (Wechsler Intelligence Scale for Children III) was 6 months before her first psychiatric appointment and reported an IQ of 114 (verbal IQ, 106; performance IQ, 121).

Her first psychiatric consultation occurred before the first COVID-19 outbreak in Portugal. Her mother was highly concerned about a recent life event: the patient's father had suffered a myocardial infarction complicated with hypoxic ischemic encephalopathy 15 days before and was, since then, hospitalized in an intensive care unit.

The patient lived with her parents and her two sisters, with limited social and extended family support as her father's family was from another region of the country and her mother's family was from another continent. Her father was the family's primary financial provider.

The patient was teased by her classmates about her body changes, weight gain, and attention difficulties associated with her medication side effects, making her sad, which also worried her mother.

During clinical observation, the patient was well-groomed and dressed appropriately to her age. Her speech was spontaneous, fluent, and age appropriate, mostly about past episodes when she felt rejected or was teased by her classmates. She presented difficulties regarding emotional states' recognition and their expression. There were no psychotic symptoms, and she denied sleep or appetite disturbance.

In the following 6 weeks, during psychiatric appointments, the patient was hesitant to talk about her fathers' condition, diverting the conversation to other daily problems as an avoidance behavior.

We aimed to provide a safe environment for emotional expression and exploration of negative and positive coping mechanisms. As the patient liked and felt competent expressing herself through drawing, we used this strategy in consultations and proposed drawing as a mean to express and share her feelings with others.

We explored her ambivalence about visiting her father, who had been transferred to internal medicine service meanwhile. At this point, her father remained in minimally conscious state and suffered multiple nosocomial infections, with poor prognosis of recovery.

During consultations, the patient told us about a fantasy she had that her father was not in the hospital, but he would be traveling and would return well and safe. Visiting the father, after careful preparation of all the involved (the patient, her mother, child psychiatrists, and father's medical team), would be helpful in reducing anxiety and regaining some sense of control. She was also concerned about the possibility of her father being in pain or discomfort, so contacting the medical and nursing team who could explain the care and treatment her father was receiving would reassure her.

We arranged meetings alone with the mother to explore her concerns and to provide psychoeducation about normal grief reactions, as well to normalize the temporary reduction in patient's academic performance. Those interviews with the mother alone were crucial to discuss the importance of giving information about the father's clinical state incrementally in small doses, in an optimistic but not unrealistic way.

We were planning a hospital visit, preparing all the involved when the government declared suspension of visiting arrangements at all hospitals and nationwide lockdown.

In the next weeks, follow-up was conducted by telephone calls.

Adding to the distress about her father's health, the challenges related to home schooling, social contacts' restriction, suspension of extracurricular activities, and change in family routines caused increased anxiety in all family members.

The father died 2 weeks after emergency state was declared in Portugal. Although his death was not COVID-19 related, the funeral obeyed the norm published at beginning of the pandemic by the Directorate-General of Health regarding postmortem and mortuary care. Only the mother and an uncle of the patient attended the funeral. The patient, her sisters, and their mother organized a ceremony at home, where they lit a candle near photographs of the family and the father and shared thoughts and feelings about him.

Clinical interviews were maintained by telephone calls once every 2 weeks and by face-to-face appointments monthly. The patient was tearful, sad, and complained about concentration difficulties during online classes. There was a decline in the patient's school grades, which was a high concern for her mother. The patient also complained about episodic initial insomnia that spontaneously remitted. The mother's emotional well-being and change in familial roles and responsibilities were also explored. The death of the patient's father implied a change in the family income, and consequently, she had to attend a different school, which made her anxious.

She gradually verbalized feeling sad and anxious about the future without her father, the family's financial situation, and her mother and sisters' well-being.

Strategies to help the patient and her family to adjust to the new reality and adaptative ways to remember the deceased were shared. The patient was encouraged to choose a linking object as memento. Besides, she started sleeping with a stuffed animal her father gave her years before, which provided a way to externally maintain a relationship with her deceased father. In the next months, she learned how to sew and knit and made a t-shirt to the stuffed animal. As the patient and her mother recalled pleasant and funny experiences with the deceased during consultations, it was suggested to make a memory book as a family activity. It would include family stories, traits the family missed about the deceased, photographs, writings about activities family engaged together, drawings, etc.

## Discussion

The death of a parent is an extremely stressful life event and is associated with increased psychiatric problems in the short and long term. The normative sadness and suffering in the grieving process can develop into psychopathology requiring mental health professional intervention. Previous studies report higher rates of depressive symptomology in bereaved children compared to community controls (15–17).

Cerel et al. interviewed 360 children (6–17 years old) and their surviving parents 2, 6, 13, and 25 months after parental death and compared them to 110 depressed children and 128 community control children. Bereaved children showed more behavioral, anxiety, and depressive symptoms than community controls in the first 2 years, although less than clinically depressed children (16). While in most bereaved children, depressive symptoms gradually decline over time, the risk for depression remains higher than community controls up to 2 years after the death (16–18). Parental death has also been associated with increased risk for posttraumatic stress disorder (17); alcohol or substance abuse (18); and lower competence in peer relations, work, career planning, and educational aspirations (19).

Death circumstances related to COVID-19, quarantine policies, and social–economical stressors can affect all necessary tasks for an adaptative grieving process. The number of individuals and families struggling with complicated grief is expected to increase during and after the pandemic (20–22).

Kentish-Barnes et al. evaluated relatives of patients who died on ICU and identified symptoms of complicated grief in 52% of them. Among the risk factors for complicated grief were patient dying while intubated, relatives not being present at the time of death, relatives not saying goodbye to the patient, and inadequate communication between physicians and the relatives (23), which are frequent in deaths related to COVID-19.

Time criteria for the diagnosis of Prolonged Grief Disorder (6 months) and Persistent Complex Bereavement Disorder (12 months; 6 months in children) impede those diagnoses among individuals who lost a loved one during COVID-19 pandemic. Eisma et al. compared acute grief levels among people recently bereaved due to COVID-19, natural, and unnatural causes, considering that acute grief is a strong predictor for pathological grief. Those who lost someone due to COVID-19 reported more severe acute grief reactions than those who lost someone to natural causes (but not unnatural causes) (24).

Accepting the reality of the loss is crucial in the mourning process. Funerals and ceremonies help the grieving process, as they make the loss real and final, get the social support network close and allow family and friends to share feelings about the deceased (6). Therefore, COVID-19 protection measures, such as hospital visits suspension and restrictions on funerals deprive family members of a proper farewell. Rules of social distancing, closing cemeteries, and prohibiting religious celebrations and other worship events do not allow friends and family to express their support and affection, leaving the beavered with feelings of loneliness.

Children inclusion in decisions about the funeral and ceremonies increase their sense of involvement and reduce their perceived sense of lack of control, empowering them (6, 25, 26). They should be given the choice to attend the funeral, and their involvement can be accomplished in multiple degrees, such as deciding what objects to put in the coffin, including them when choosing the funeral flowers or music, letting them help selecting the tombstone or even helping carrying the coffin (25). The degree of their involvement depends on their developmental stage, cultural, and religious customs. If they choose to attend the funeral, it should be explained in advance and in age-appropriate terms what may happen and what they might see. They need to be protected from strong emotions that may be displayed. Therefore, it is helpful to let them be accompanied, by someone they like, but who is not close to the deceased, and with who they can leave the funeral if necessary (6, 27).

With the COVID-19 pandemic, direct participation of children and other family members in the formalities is more difficult. As their inclusion is of most importance, we should consider their participation in indirect ways, such as asking them to write something they want to be said during the funeral, describing the ceremony to them in a sensitive and appropriate way, participating in family rituals, and respecting their opinions and decisions about cemetery visits.

Reestablishment of family routines is a known protective factor in the bereavement process, and it may be problematic because of measures imposed by the COVID-19 pandemic, such as school closures and telework (1, 6).

Additionally, we should consider the variety of grief manifestations in children. Even if they do not disclose their emotions right away, they might begin to make the connection between emotions and how their body is reacting, grieving with complains of physical symptoms. A visit to the pediatrician or neuropediatrician may be advised in some cases, to reassure the child that nothing is wrong. On the other hand, children often express their emotional discomfort with anger, which should be address so it does not escalate or feed on itself. Physical activity and physiotherapy have been consistently associated with activation of multiple neurophysiological processes involved in discomfort relief and mood improvement (28, 29). As bereavement also interferes in occupations and occupational performance of bereaved child, the occupational therapist should also be included in support teams providing spaces of speech, resignification, and reflection to reduce occupational losses (28, 30). Therefore, grief intervention in children should be carried out by a coordinated multidisciplinary team comprising healthcare professionals from different disciplines, who work in regular collaboration with one another and with schools and families to ensure the provision of consistent, goal-directed care (30). However, multidisciplinary work to help children regain educational, recreational, and social skills is hampered by school and extracurricular activities suspension, meetings and consultations restrictions, and other COVID-19 protection measures.

Among the risk factors that have been associated with higher risk of psychopathology in parent-bereaved children are parental suicide (18), the surviving parent's level of depression, family socioeconomical status, and the presence of other stressful life events in the family (16). Additional to their own overwhelming grief and distress, the surviving parent may have to deal with bureaucratic constraints imposed by restrictions on public services and with the economic impact of the COVID-19 pandemic. As the functioning of the surviving parent and family relationship and communication patterns are important mediators of the grief process in children who lost a parent (26), intervention programs for bereaved children should also include the surviving parent. Interventions directed to the surviving parents and/or the family reduce maladjusted grief responses years after the loss (31, 32).

Grief counseling and therapy for those struggling with the grieving process had to adapt to the new reality that COVID-19 pandemics brought us. Hospitals had to quickly reorganize services, and non-urgent surgeries and face-to-face consultations were suspended. Individual and family psychotherapeutic interventions had to be held by telephone or videoconference.

Before the pandemic, child, and adolescent telepsychiatry was mostly used to address the disparity in access to services for populations with inadequate psychiatric care and to provide care in non-traditional settings, such as schools, correctional facilities, and at home (33).

Although psychiatric teleconsultation is essential during COVID-19 pandemic, as it allows to maintain contact with patients and to help them in a safely manner, it presents several limitations. The abrupt switch to telemedicine, the impossibility to capture the patient's non-verbal communication clues through telephone calls, and the physical and affective distancing while dealing with sensitive issues such as death may represent a burden in the therapeutic relationship.

Other obstacles must be considered, namely, technological difficulties for patients and clinicians. Such problems are especially important in psychiatric services, where families may struggle with financial hardship, which can complicate access to technology. Maintaining privacy and confidentiality can be worrisome, especially for large families living in small houses, where it can be difficult to find an isolated place to talk openly and freely with the psychiatrist (34–36).

Wagner et al. conducted a systematic review and meta-analysis regarding web-based interventions (based on cognitive behavioral therapy) for people with elevated levels of disturbed grief. Their results suggest that this type of intervention can help in reducing symptoms of posttraumatic stress disorder and grief (37).

## Conclusion

Mourning a parent during childhood or adolescence is stressful enough *per se*. Going through this process during the COVID-19 pandemic, with its uncertainties and rapid changes in daily life, can be a real struggle.

Child and adolescent psychiatrists as well as other mental health professionals should be prepared to help the numerous individuals and families who are expected to struggle with complicated grief, depression, and posttraumatic stress disorder. Knowing and comprehending the grief process and developing and disseminating grief counseling and treatment programs possible to be remotely delivered should be a priority not only for clinicians but also for policy makers. Policies and rules regarding the COVID-19 pandemic have to considerate measures to protect mental health, facilitating the grief process.

## Data Availability Statement

The original contributions presented in the study are included in the article/supplementary material, further inquiries can be directed to the corresponding author/s.

## Ethics Statement

Written informed consent was obtained from the individual(s), and minor(s)' legal guardian/next of kin, for the publication of any potentially identifiable images or data included in this article.

## Author Contributions

SS and TS conducted the patient's consultations under the supervision of IA, IC, ZC, and TC. SS conducted the literature search and wrote the first draft of the manuscript. SS and TS wrote the final version of the manuscript. IA, IC, TC, and ZC critically revised the manuscript.

## Conflict of Interest

The authors declare that the research was conducted in the absence of any commercial or financial relationships that could be construed as a potential conflict of interest.
